# The role of the annexin A protein family at the maternal–fetal interface

**DOI:** 10.3389/fendo.2024.1314214

**Published:** 2024-03-01

**Authors:** Jingwen Hu, Lin Chen, Jing Ruan, Xiaoyan Chen

**Affiliations:** ^1^ Maternal-Fetal Medicine Institute, Department of Obstetrics and Gynaecology, Shenzhen Baoan Women’s and Children’s Hospital, Shenzhen University, Shenzhen, China; ^2^ Fertility Preservation Research Center, Department of Obstetrics and Gynaecology, The Chinese University of Hong Kong, Hong Kong, Hong Kong SAR, China

**Keywords:** annexin family, calcium and phospholipid binding proteins, maternal-fetal interface, trophoblast, female reproductive disorder

## Abstract

Successful pregnancy requires the tolerance of the maternal immune system for the semi-allogeneic embryo, as well as a synchrony between the receptive endometrium and the competent embryo. The annexin family belongs to calcium-regulated phospholipid-binding protein, which functions as a membrane skeleton to stabilize the lipid bilayer and participate in various biological processes in humans. There is an abundance of the annexin family at the maternal–fetal interface, and it exerts a crucial role in embryo implantation and the subsequent development of the placenta. Altered expression of the annexin family and dysfunction of annexin proteins or polymorphisms of the *ANXA* gene are involved in a range of pregnancy complications. In this review, we summarize the current knowledge of the annexin A protein family at the maternal–fetal interface and its association with female reproductive disorders, suggesting the use of ANXA as the potential therapeutic target in the clinical diagnosis and treatment of pregnancy complications.

## 1 Introduction

The maternal–fetal interface is a crucial site for the establishment and maintenance of normal pregnancy, where the trophoblast cells, decidual cells, and the immune microenvironment exist and interact with each other. Dysregulation of the functions of the trophoblast and disturbance of the maternal–fetal immune tolerance can lead to reproductive disorders, including infertility, spontaneous miscarriage, preeclampsia, and intrauterine growth restriction.

Annexins, a well-known multigene family, are secreted proteins in the cytoplasm attaching to the phospholipid membrane and highly conservative Ca^2+^-dependent membrane-binding proteins that participate in a variety of physiological and pathological processes in humans (1–3). The role of the annexin family participating in several human pathologies such as tumorigenesis, obesity, and atherosclerosis has been extensively reviewed in a bunch of excellent papers (4–7). The expression of the annexin family was identified at the maternal–fetal interface, involving both the trophoblasts and decidual cells, but its role in successful pregnancy is not fully clear yet. Herein, we reviewed the current knowledge of the expression and possible functions of the annexin family at the maternal–fetal interface and its relationship with female reproductive diseases.

## 2 The annexin family

Annexin is an evolutionally conserved Ca^2+^-regulated phospholipid-binding protein superfamily, which is named for its ability to “annex,” implying that it integrates membranes, and is widely distributed in eukaryote cells (8). To date, more than 1,000 members of the annexin superfamily have been identified and classified into five groups (groups A–E) according to different species, with *ANXA12* being a pseudogene. The annexin A group comprises 12 different members (AnxA1–AnxA11, AnxA13) in human organs. Annexins participate in extracellular activities including proinflammatory and profibrotic responses, as well as subcellular functions consisting of membrane repair, cytoskeletal changes, intracellular organelle transportation, and signal transduction. Over the past decades, a great number of studies on annexins have been performed for their involvement in a variety of physiological and pathological processes in humans, among which AnxA1 and AnxA5 are in the process of development as potential therapeutic targets for atherosclerosis, fibrosis, rheumatoid arthritis, and cardiovascular disease currently (9–12).

### 2.1 Molecular structure of the annexin family

The 12 human annexin genes are dispensed throughout the genome on chromosomes 1, 2, 4, 5, 8, 9, 10, and 15, with the genomic size ranging from 15 kb (*ANXA9*) to 96 kb (*ANXA10*). The expression levels of the annexin gene are very wide and present different abundance levels in specific tissues (13).

**Figure 1:**
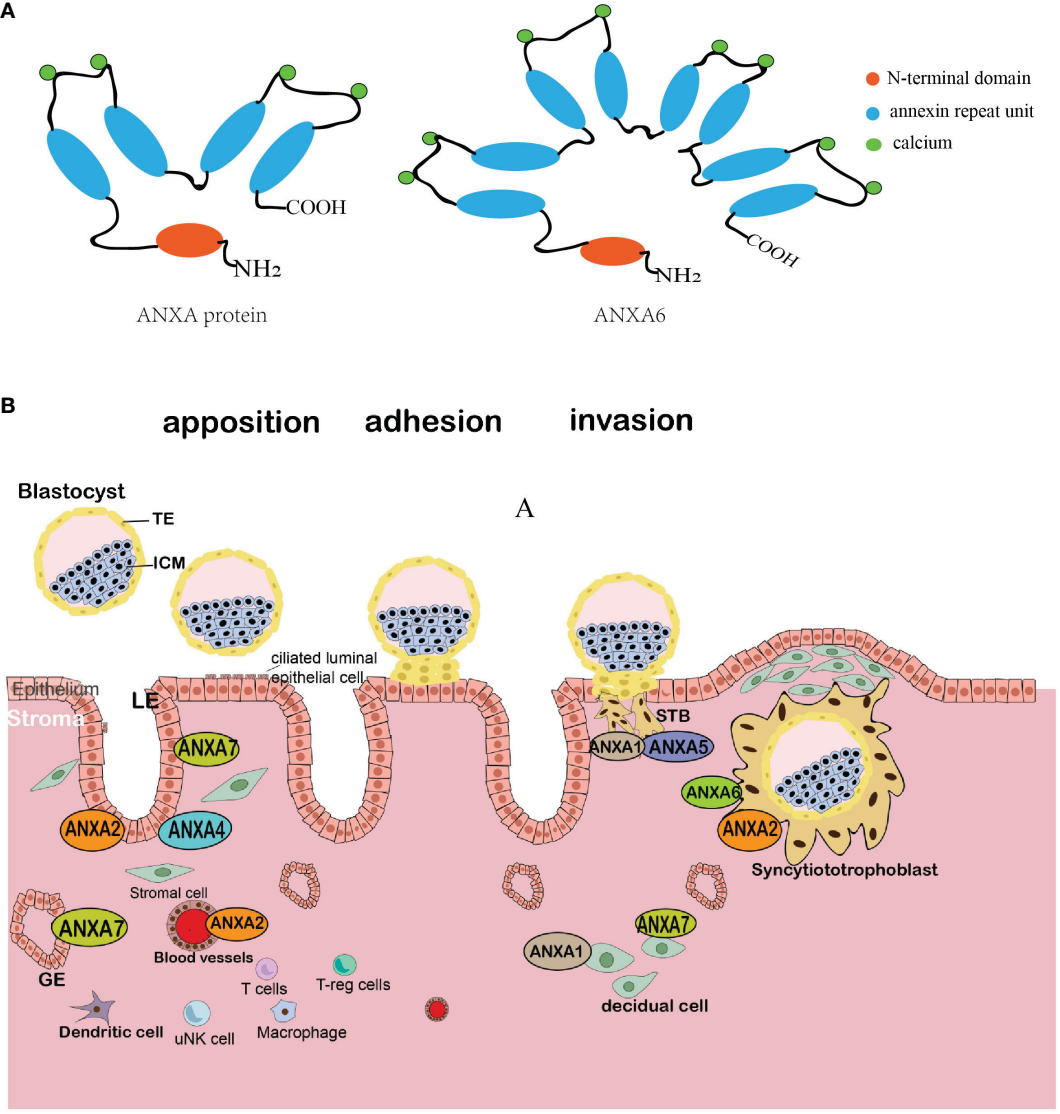
Schematic diagram of the annexin molecular structure and comprehensive schematic illustration of the location of the annexin family at the maternal–fetal interface. **(A)** The ANXA protein structure comprises two main regions: a highly conserved C-terminal core with four repetitive sequences (or eight in ANXA6), each harboring a calcium-binding motif, and a variable N-terminal with differing amino acid arrangements and lengths across each ANXA variant. **(B)** The blastocyst implants to the maternal endometrium following the sequential stages of apposition, adhesion, and invasion. Members of the annexin family show different expression patterns in diverse cell types as the blastocyst interacts with the endometrium. TE, trophectoderm; ICM, inner cell mass; LE, luminal epithelium; GE, glandular and luminal endometrial epithelium; STBs, syncytial trophoblasts; uNK, decidual natural killer cell.

Structurally, the protein of the annexin A family consists of two domains: the conserved C-terminal and the variable N-terminal (14). All annexins share the conserved C-terminal domain, which is a highly helical and tightly folded protein center consisting of four (or eight in AnxA6) annexin repeat units; thus, a characteristic “type 2” calcium-binding site is formed through hydrophobic interactions and ensures structural stabilization (15). In contrast, unlike the core domain, the N-terminal (also known as the tail domain) is a variable domain containing sites for post-translational modification and is unique in each member, enabling these members to bind to various ligands and other proteins; thus, the members present a diversity of locations and functions (16) (**Figure 1A**).

In most circumstances, annexins bind reversibly to cell membranes via the featured calcium-binding site. Another common scenario, as mentioned above, the unique N-terminal domain, mediates the interaction between annexins with other specific intracellular protein partners, such as the S100 family cytoplasmic proteins. The S100 protein is a dimeric EF-hand calcium-binding protein and forms a protein–protein interaction with the N-terminal domain of AnxA2 through a highly specific binding site, thus collaborating to regulate multiple cellular functions, leading to the AnxA2/S100A10 complex possessing biological activities (17–19).

### 2.2 Functions of annexins

Annexins are involved in several cellular membrane-related processes, including cellular structural organization, growth regulation, and vesicle transportation. To date, the relationship between annexins and various cellular processes including vesicle trafficking, membrane repair, cell proliferation and apoptosis, cell migration, anti-inflammation, promotion of angiogenesis, and anticoagulation has been reported in *in-vitro* gain-of-function or loss-of-function experiments, and these processes are independent of calcium signaling. On a pathologic front, accumulating evidence indicates that annexins exert essential roles in the pathogenesis or progression of numerous human diseases, including various types of cancer, autoimmune disorders, diabetes, and cardiovascular diseases.

The expression patterns of annexins differ significantly in various organs with high expression patterns found in the smooth muscle, thymus, and lung and relatively low expression patterns in the testis, adrenal glands, and brain. Interestingly, the expression of annexins could show a dynamic change along with the cell cycle, which may be associated with the microtubule, vesicle transportation, and Ca^2+^-regulated exocytosis in tissues undergoing myriads of cellular processes or mechanical changes throughout the body (20). As the annexin family is abundantly expressed at the maternal–fetal interface, and both the endometrium and blastocyst undergo rapid growth and differentiation during pregnancy, it is believed to play a series of roles both in the fetus-derived placenta and maternally derived decidual cells.

## 3 The annexin A protein family at the maternal–fetal interface

### 3.1 The establishment of the maternal–fetal interface

The fetus-derived trophoblast cells and maternal decidua constitute the maternal–fetal interface. On the fetal side, when the embryo implants completely into the endometrium, the trophoblast is the epithelial overlying layer of the fetal villi floating in the maternal blood, where the trophoblast proliferates and differentiates into cytotrophoblast cells (CTBs). CTBs further differentiate into the syncytiotrophoblasts (STBs) and the extravillous trophoblasts (EVTs). The syncytial trophoblast serves as the outer layer of the placental villi, which directly contacts with decidual glandular secretions and participates in the exchange of nutrients, gases, and waste products between maternal and fetal blood. Furthermore, the STBs dominate the synthesis and secretion of hormones. On the maternal side, the decidual tissue is stimulated by the decidualization inducer and transformed from the endometrium, consisting of luminal epithelial cells, glandular epithelial cells, endothelial cells, differentiated stromal cells, and a diversity of immune cells. Decidual stromal cells (DSCs) are dominant components of the decidua and secrete a wide range of factors for the regulation of the microenvironment at the maternal–fetal interface during early pregnancy (21). In addition, immune cells accumulate and endometrial arteries become more distorted and elongated during decidualization (22). In humans, the endometrium undergoes pre-decidualization as it transforms into the secretory phase during every menstrual cycle, thus allowing the embryo to attach and penetrate (23).

### 3.2 The annexin A protein family at the maternal–fetal interface

#### 3.2.1 Annexin A1

Annexin A1 (AnxA1) (also known as lipocortin-1 or p35) is a glucocorticoid-induced protein extensively researched as a potent anti-inflammatory molecule in the periphery (24). The expression of AnxA1 in the placenta is mainly in the cytotrophoblast cell surface and syncytial knots as well as in decidual cells (25, 26). A recent study has reported that AnxA1 inhibits inflammation to maintain the optimal microenvironment for implantation (27, 28). Furthermore, AnxA1 was found to be associated with the dynamic interaction between the uterine epithelium and vascular endothelium, a crucial process for successful and adequate decidualization (29).

#### 3.2.2 Annexin A2

Annexin A2 (AnxA2) is ubiquitously expressed and presents an abundance typically in endothelial cells and monocytes, and the upregulation of AnxA2 expression is observed in tumor cells and regarded as a marker of multiple tumors (30). AnxA2 was also upregulated in human receptive endometrium, especially in the luminal epithelium (LE) (31), which was an essential intracellular protein for embryo attachment (32). Matorras et al. have also reported decreased levels of AnxA2 in human endometrial fluid aspirated during the pregnancy cycle compared with the unconceived cycle (33). Another study further proved the function of AnxA2 in the process of decidualization (34). Furthermore, both estrogen and progesterone at physiological levels increase the overall expression of *ANXA2* in human endometrial cells (35). Meanwhile, using proximity ligation technology, researchers have observed the precise *in-situ* location of AnxA2/S100A10 complexes in the human placenta, which showed high levels of expression of the complexes in the amniotic membrane and vascular endothelium cells; however, the expression level was lower in the brush border region of syncytial and trophoblast cells (36).

#### 3.2.3 Annexin A4

Annexin A4 (AnxA4) is predominantly expressed in epithelial cells and reported to increase in epithelial cell tumor (37). AnxA4 is among the most intensively studied proteins in the human endometrial proteome, which is located in the glandular and luminal epithelium and has an impact on endometrial functions via the regulatory effect of ion and water movement across the membrane (38).

#### 3.2.4 Annexin A5

In humans, annexin A5 (AnxA5) is a protein that is most abundantly expressed in the placenta, and its local anticoagulant function has been well described (39). AnxA5 is highly expressed on the apical surfaces of STBs and exerts a crucial role in maintaining blood flow for placental circulation. It has recently been reported that AnxA5 is important in successive steps of membrane overlap and cohesion in both human cytotrophoblasts and STBs (40). AnxA5 presents a cyclical change in the uterine cycle and is found to be upregulated in the luteal phase endometrium in healthy fertile women (41, 42). Meanwhile, the antiphospholipid antibody could mediate the crystallization destruction of AnxA5 on the phospholipid bilayers and cell membranes including endothelial cells and trophoblast cells, accounting for the pathology in antiphospholipid syndrome (43, 44).

#### 3.2.5 Annexin A6

The dual-core annexin A6 (AnxA6) is predominantly found at the plasma membrane and endosomal compartment (45, 46). As a well-known plasma membrane repair protein, AnxA6 acts as a multifunctional scaffolding protein and interacts with phospholipid membranes and different signaling proteins (15, 47). One earlier study has reported that AnxA6 is expressed in the apical and basal STB membranes and could regulate the Maxi-chloride channel in the human placenta (48).

#### 3.2.6 Annexin A7

Annexin A7 (AnxA7) is the first member of the annexin family proteins to be discovered in humans. ANXA7 is expressed in the endometrial glands, stroma, and luminal epithelium according to the *Human Protein Atlas* (49). The expression of ANXA7 was found upregulated during the process of decidualization, which indicated its conserved role in regulating endometrial receptivity and embryo implantation (50).

#### 3.2.7 Annexin A8

Annexin A8 (AnxA8), also known as vascular anticoagulant-beta 1 (VAC-beta), is expressed at a smidgen level in the placenta, liver, cornea, and lungs (51, 52). The protein is a minor component in the placenta, accounting for less than 1% of all extracted annexins (53). The role of AnxA8 in regulating the proliferation of endometrial cells was observed in porcine (54). However, whether AnxA8 plays a similar role in human endometrium remains unclear.

## 4 The annexin family and female reproductive disorders

Disruptions of the components at the maternal–fetal interface could lead to placenta dysfunction, including impairing trophoblast invasion function, hindering angiogenesis in the uterus, affecting the process of decidualization, and compromising maternal–fetal immune tolerance (55, 56). Such disturbances are implicated in a spectrum of pregnancy-related complications. Within the human body exists a regulatory network characterized by both precision and complexity, and many mechanistic experiments are conducted utilizing some specific cell lines *in vitro*. Many antibodies and molecules existing in the peripheral blood play a unique role at the maternal–fetal interface. For instance, research has shown that antiphospholipid antibodies (aPLs) interacting with β2-glycoprotein I (β2GPI) modulate the expression of Bcl-2 and Bax proteins in primary human trophoblasts (57). Furthermore, the influence of anti-transglutaminase type 2 (anti-TG2) autoantibodies on endometrial angiogenesis has been explored using human endometrial endothelial cells (HEECs), shedding light on potential pathogenic mechanisms underlying placental damage in celiac disease (58). Additionally, recent findings indicate the presence of HLA-DR in STBs and STB-derived extracellular vesicles (STEVs) in a significant number of preeclampsia cases, as opposed to control placentas, suggesting a novel avenue of investigation in placental pathology in preeclampsia (59). Alterations in the expression levels of the annexin A proteins are also proven to interrupt those key processes in the establishment of pregnancy.

### 4.1 Infertility and recurrent implantation failure

In human-assisted reproductive technology (ART) programs, more than 60% of women treated with *in-vitro* fertilization (IVF) procedures fail to achieve clinical pregnancy after their first transfer and almost 20% of them suffer from unexplained recurrent implantation failures (RIFs) (60). RIF can be defined as failure to clinical pregnancy in a woman under 40 after the transfer of at least four good-quality embryos in at least three fresh or frozen cycles (61). The protein S100-A10 (S100A10), a binding partner of AnxA2, was identified as a critical factor in endometrial receptivity attainment and was downregulated in the mid-secretory phase of the endometrium of infertile women (62). Recent studies have reported that maternal and paternal M2/ANXA5 haplotype carriages are both risk factors for RIF, which shed light on the pathogenesis of RIF and provided possible forecasts for couples who are at a pertinent risk ahead of the ART programs (63).

### 4.2 Recurrent pregnancy loss

Recurrent pregnancy loss (RPL) is defined as at least two or three spontaneous miscarriages before the 24th gestational week and impacts approximately 1%–3% of reproductive-age women (64). Anatomical abnormalities, endocrine disorders, genetic factors, and immunological factors are considered responsible for RPL; however, almost half of the patients suffered for unexplained reasons (65–67). The role of AnxA5 in RPL has been extensively investigated since Rand JH et al. found that the level of AnxA5 and its anticoagulant activity are significantly reduced in the plasma of RPL patients (68). Moreover, the variants of *ANXA5* in the placenta are found important in the pathology of RPL. The haplotype M1 is defined as the combination of two alleles, namely, c.−448 ANC and c.−422 TNC, and reveals single nucleotide polymorphisms (SNPs) of 1A/C and 27T/C. The M2 haplotype, on the other hand, is identified as having four small alleles corresponding to these four SNPs, rs112782763 (c.−467 GNA), rs28717001 (c.−448 ANC), rs28651243 (c.−422 TNC), and rs113588187 (c.−373 GNA), which contains a combination of SNPs of 19G/A, 1A/C, 27T/C, and 76G/A and can be passed on to the offspring (69, 70). Several independent studies have shown that the M2 haplotype in *ANXA5* has been linked to greater overall RPL risk mostly for early miscarriage, ranging between the 10th and 15th gestational weeks (71–73). A meta-analysis of 14 independent retrospective case–control studies also summarized that *M2/ANXA5* haplotypes in couple populations have a significantly higher risk for RPL in comparison to the normal haplotype (74). Antiphospholipid syndrome (APS) is characterized as an autoimmune disorder that predominantly manifests in thrombotic events. It is observed that approximately 6% of patients with APS experience complications related to pregnancy (75). Extensive research indicates that antibodies in patients diagnosed with APS impede the crystallization and anticoagulant function of AnxA5. This interference leads to a diminished response to the anticoagulant properties of AnxA5 (43). Furthermore, it has been established through *in-vitro* studies that the application of anti-annexin V monoclonal antibodies (mAbs) precipitates apoptosis in trophoblast cells and results in a marked decrease in human chorionic gonadotropin (hCG) secretion (76).

### 4.3 Preeclampsia

Preeclampsia (PE) refers to new-onset hypertension, proteinuria with maternal multi-organic dysfunction, or fetal growth restriction after the 20th gestational week (77). PE is the leading cause of maternal and perinatal mortality, occurring in approximately 5% of pregnancies (78, 79). However, the pathological mechanism of PE remains obscure. It is believed that PE is related to shallow invasion of the trophoblast and poor placental perfusion at the maternal–fetal interface, as well as maternal vascular endothelial injury and vascular endothelial dysfunction accompanied by maternal systematic inflammation (56, 80). A recent study has reported that modulation of AnxA1 in the trophoblast is associated with systemic inflammatory response-related preeclampsia (81). Using the 2D-PAGE technique, Gharesi-Fard Behrouz et al. (82) found that AnxA1, as a placental protein, is increased in PE patients, indicating exacerbated systemic inflammation in PE (83). One earlier study has reported that the AnxA2 protein both in the placenta and peripheral maternal blood was downregulated significantly in patients with PE compared with normal pregnancies, which was linked to microvascular thrombin formation in PE (84). Defects in decidualization are also considered as the maternal factor of preeclampsia (85, 86). Researchers further found that defective expression of endometrial *ANXA2* might impair the decidualization of endometrial stromal cells *in vitro* and *in vivo*, and inhibition of *Anxa2* in mice failed to support embryo invasion *in vivo* functionally (32, 87). Xu et al. demonstrated that *ANXA4* expression is downregulated in human placentas in PE, and *ANXA4* overexpression in human trophoblast cells may promote trophoblast invasion via the PI3K/AKT/eNOS pathway (88). Another systematic review, the first to combine proteomic studies of the placental biopsies of PE and polycystic ovary syndrome, found five biomarkers for PE which are common in women with PCOS, among which AnxA4 was downregulated in both groups of patients (89). In addition, the M2 haplotype of *ANXA5* was also observed to be more prevalent in the placenta of women with PE compared with the controls, which may significantly increase the risk for PE by impairing the thrombomodulatory function of AnxA5 at the maternal–fetal interface (90, 91).

### 4.4 Intrauterine growth restriction

Small for gestational age (SGA) is defined as the birth weight of a newborn less than the 10th percentile for the corresponding gestational age (92). SGA fetuses are diagnosed with intrauterine growth retardation (IUGR) if they fail to achieve their genetically determined growth potential at any gestational age. Intrauterine growth restriction (IUGR) affects 10%–15% of all pregnancies worldwide (93). IUGR may result from maternal, placental, or fetal factors (94).

Earlier studies have reported that AnxA5 is present in the amniotic fluid and increased during 15 to 24 weeks of gestation. AF-Anxa5 levels are elevated in patients who develop IUGR, which indicates AF-AnxA5 a potential marker for identifying IUGR (95, 96). Another study further reported that decreased *ANXA5* mRNA levels were detected in the placenta from SGA pregnancies in comparison to normal outcomes (97). Moreover, a recent study has demonstrated a significantly higher prevalence of the M2 haplotype in women who have delivered an SGA fetus (98), which coordinated with the extensively reported dysfunction of the *ANXA5* haplotype in RPL and pre-eclampsia.

## 5 Conclusion

The expression of annexins in the maternal–fetal interface suggests their roles in embryo implantation and pregnancy. For the maternal side, the expression levels of AnxA1, AnxA2, AnxA4, and AnxA7 are found in decidual stromal cells and epithelial cells, and AnxA1, AnxA2, AnxA5, and AnxA6 are expressed in the trophoblast, especially in the apical and basal STB membranes (**Figure 1B**). In addition, there is an altered expression of annexins in women with reproductive disorders, such as recurrent implantation failure, endometriosis, adenomyosis, and recurrent pregnancy loss (**Table 1**). However, whether the annexin family can be used as clinical markers remains uncertain, and further studies are required.

**Table 1:** Summary of the role of the annexin family in female reproductive disorders.

Annexin member	Location	Biological functions and role	Related disease	Ref.
AnxA1	Glandular epithelium cells	Participate in cell differentiation and proliferation via FPR1 and FPR2 receptors	Endometriosis	(99)
AnxA1	Syncyotiotrophoblast and villous vascular endothelial cells	Attenuate the exacerbated inflammatory response	Preeclampsia	(81, 82, 100)
AnxA2	Endometrial epithelium and trophoblast	Human embryo attachment	NA	(35, 36)
AnxA2	Endometrial epithelium cells in the ectopic endometrium	1) Promote the growth, distant metastasis, and angiogenesis in AM endometrial tissue 2) Aggravate dysmenorrhea	Adenomyosis	(101, 102)
AnxA2	Syncytiotrophoblasts	Impair the placental fibrinolytic activity	Preeclampsia	(84)
AnxA2	Endometrial stromal cells	Reduce human endometrial stromal cell motility and embryo invasion	Preeclampsia	(87)
AnxA4	Endometrial tissue	Participate in the anti-apoptosis process	Polycystic ovarian syndrome	(103)
AnxA4	Trophoblasts	Promote cell migration and invasion of trophoblast cells via the PI3K/AKT/eNOS signaling pathway	Preeclampsia	(88)
AnxA5	Endometrial glands and stroma cells	Promote apoptosis	Polycystic ovarian syndrome	(103)
AnxA5	Ectopic endometrium	Inhibit apoptosis and promote migration and invasion	Ovarian endometriosis	(104)
AnxA5	Perivillous and extravillous trophoblasts	Promote the fluidity of maternal blood circulating through the intervillous space	Preeclampsia	(105, 106)
AnxA5	Apical surfaces of syncytiotrophoblasts	Shield phospholipid bilayers from exposure to coagulation reactions	Intrauterine growth restriction	(95–97)
AnxA5	Placenta villi	Play a critical role in cell membrane repair to maintain the integrity of the placenta	Recurrent pregnancy loss	(70, 107, 108)

NA, not applicable.

## Author contributions

JH: Writing – original draft. LC: Writing – review & editing. JR: Writing – review & editing. XC: Writing – review & editing.
